# RE-YOLO: An apple picking detection algorithm fusing receptive-field attention convolution and eﬃcient multi-scale attention

**DOI:** 10.1371/journal.pone.0319041

**Published:** 2025-03-03

**Authors:** Jinxue Sui, Li Liu, Zuoxun Wang, Li Yang

**Affiliations:** 1 School of Information and Electronic Engineering, Shandong Technology and Business University, Yantai, China; South China University of Technology, CHINA

## Abstract

The widespread cultivation of apples highlights the importance of eﬃcient and accurate apple detection algorithms in robotic picking technology. The accuracy of current apple picking detection algorithms is still limited when the distribution is dense and occlusion exists, and there is a significant challenge in deploying current high accuracy detection models on edge devices with limited computational resources. To solve the above problems, this paper proposes an improved detection algorithm (RE-YOLO) based on YOLOv8n. First, this paper innovatively introduces Receptive-Field Attention Convolution (RFAConv) to improve the backbone and neck network of YOLOv8. It essentially solves the problem of convolution kernel parameter sharing and improves the consideration of the differential information from different locations, which significantly improves the accuracy of model recognition. Second, this paper innovatively proposes an EMA_C2f module. This module makes the spatial semantic features uniformly distributed to each feature group through partial channel reconstruction and feature grouping, which emphasizes the interaction of spatial channels, improves the ability to detect subtle differences, can effectively discriminate the apple occlusion, and reduces the computational cost. Finally, the loss function of YOLOv8 is improved using the Wise Intersection over Union (WIOU) function, which not only simplifies the gradient gain assignment mechanism and improves the ability to detect targets of different sizes, but also accelerates the model optimization. The experimental results show that RE-YOLO improves the precision, recall, mAP@0.5, and mAP@0.5-0.95 by 2%, 2.1%, 2.7%, and 3.9%, respectively, compared with the original YOLOv8. Compared with YOLOv5, it improves 4%, 1.9%, 1.7% and 3%, respectively, which fully proves the advanced and practical nature of the proposed algorithm.

## Introduction

Apples have become a global consumer product and a major agricultural commodity [1,2]. In recent years, the trend in agricultural development has been toward mechanization and automation of production processes [3]. As a result, agricultural robots have emerged as an important component of agricultural intelligence and modernization, and are experiencing unprecedented growth opportunities [4,5]. The development of apple-picking robots has immense potential to reduce labor costs and increase productivity in the apple-growing industry, while promoting digitalization and intelligence throughout the apple production chain. However, in complex natural growing environments, apple picking algorithms must overcome problems such as dense fruit distribution, shading and light variations, and current apple picking algorithms still face the challenge of poor picking accuracy and eﬃciency [6–8].

Researchers are currently addressing the above challenges by investigating apple detection techniques [9,10]. In previous studies, scientists identified and detected fruits based on their appearance characteristics, such as color, shape, and texture [11–13]. However, most methods have been tested under specific or idealized conditions, limiting their application and eﬃciency in real-world production environments [14,15]. Jia et al, extracted 16 typical features of target apples using pulse-coupled neural networks and developed a new GAElman algorithm to detect overlapping apples with a recognition rate of 88.67%. [16]. Fan et al. used a grayscale-centered RGB color space to integrate local image features and color data to effectively differentiate apple pixels [17]. Sun et al, developed an apple target segmentation technique that integrates fuzzy set theory and a flow sorting algorithm to identify green apples in similar backgrounds [18]. Lv et al, used Otsu’s dynamic thresholding segmentation method, edge detection, and an improved RHT transform method to detect apple fruits [19]. Lin et al. developed a fruit recognition system using HSV color and geometric features to characterize the appearance of various fruits [20]. Although all of these methods can be used to detect apples, they have poor robustness and generalisation in variable natural environments, making it diﬃcult to balance real-time and accuracy requirements, and cannot meet the needs of apple-picking robots working in complex scenarios.

The wide application of deep learning-based target detection technology has fully demonstrated its excellent ability to perform target detection in complex backgrounds [21,22]. Jia et al, proposed an overlapped apple target detection model based on masked R-CNN, but it is diﬃcult to meet the real-time requirements of robots due to the fact that the size of the model is much larger than that of target detection algorithms in the YOLO series [23]. Gao et al, proposed a fast region convolutional neural network-based apple detection method for multi-category dense fruit trees, which can effectively detect occluded apples with an average accuracy of 87.9% [24]. Ji et al, described a ShuﬄeNetv2-YOLOX-based apple detection method that achieves a detection speed of 26.3 frames per second on the Jetson Nano platform [25]. Yan et al, proposed an improved YOLOv5s-based target detection algorithm for apple-picking robots, redesigned the bottleneck cross-level part (CSP) module as the bottleneck CSP-2 module, and added the squeeze and excitation modules from the visual attention mechanism to the improved backbone network, with an average detection accuracy of 86.75% [26]. Xu et al, used the lightweight GhostNet as the YOLOv4 backbone network and introduced the Mish activation function in the neck network to reduce the model parameters, and the final model accuracy was improved by 2.26%, while the model size was reduced from 250.7 MB to 43.5 MB [27]. Liu et al, proposed a YOLOv5s-BC algorithm for apple detection with the addition of a coordinate attention module and a bidirectional eigenpyramid network, and the average accuracy reached 88.7% with a frame rate of 55 frames/sec and a weight size of only 16.7 MB [28]. Shen et al, fused Convolutional Block Attention Module (CBAM) and Generalised Intersection over Union (GIOU) loss function on top of the YOLOv3 model, and finally obtained a model size of 64.3M, giga floating-point operations per second is 79.5, which is diﬃcult to meet the deployment requirements on resource-limited picking robots, and the authors also suggested in the conclusion that the new version of the algorithm will be more suitable for mobile devices [29]. Tao et al, based on YOLOv5, fused PixelShuﬄe and Receptive-Field Attention (PSRFA), Multi-scale and Eﬃcient (MSE), Multi-scale and Eﬃcient (MSE), and Eﬃcient Intersection over Union (EIOU) loss functions with an average accuracy of 88% and Giga Floating-point Operations Per Second of 53.8 [30]. These studies and advances imply that deep learning is changing the face of apple farming, reducing the dependence on manual labour and accelerating the process of agricultural automation.

In summary, deep learning based apple picking detection algorithms still face many challenges, especially in terms of balancing high accuracy with real-time performance. Existing methods have not been able to effectively solve these problems and are still insuﬃcient for applications in complex natural environments. To address these bottlenecks, this paper proposes a new detection algorithm, RE-YOLO, to overcome the limitations of current techniques. The main contributions of this paper are:

(1) To overcome the limitations of traditional convolutional networks in terms of parameter sharing and to improve the detection ability of key region features, this paper innovatively introduces the Receptive-Field Attention Convolution strategy. By adding the Receptive-Field Attention mechanism to the standard convolutional layer, the feature extraction ability of the model for the difference information of different image regions is greatly enhanced, thus significantly improving the recognition accuracy.
(2) An innovative EMA_C2f module is proposed to replace the C2f module in the YOLOv8 network. This module is able to capture both near and far image features in scenes with dense targets or the presence of occlusion, which significantly improves the recognition eﬃciency and accuracy of the model. This improvement enhances the responsiveness of the model and effectively increases the overall performance of the algorithm.
(3) Aiming at the accuracy and robustness of multi-scale target detection, an improved WIOU loss function is proposed in this paper. This loss function significantly improves the performance of the model in multi-scale target detection by introducing an optimised gradient gain allocation mechanism, which further accelerates the model optimisation process.

The rest of the paper is structured as follows: The Related Works section describes the algorithms related to the base model and the methods of data collection and processing. The Methods section describes the proposed RE-YOLO model and its components in detail. The Experimental and Results Analysis section describes the evaluation metrics, the experimental setup, and the comparative experimental results. The Conclusions section discusses the state of the art and outlines future research directions.

## Related works

### YOLOv8 overview

YOLO (You Only Look Once) is a groundbreaking target detection algorithm proposed by Joseph Redmon in 2015 [31]. It has the advantage of combining accuracy, lightweight design, and scalability. As an end-to-end deep neural network, YOLO formulates object detection as a unified regression and classification problem that directly translates raw image input into the identification and localization of objects in the image.

**Fig 1:**
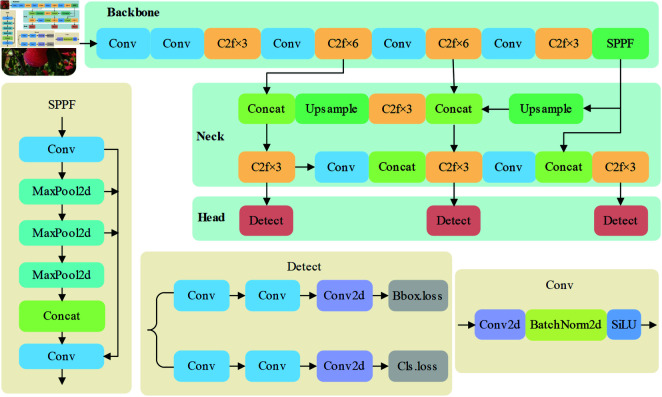
YOLOv8 network structure.

YOLOv8 is the latest version of the YOLO family released by Ultralytics in 2023 with the network architecture shown in Fig 1 [32].Compared to other versions YOLOv8 better balances accuracy and speed and has the potential for real-time detection. Therefore, YOLOv8 is used as the baseline model in this paper.

**Fig 2:**
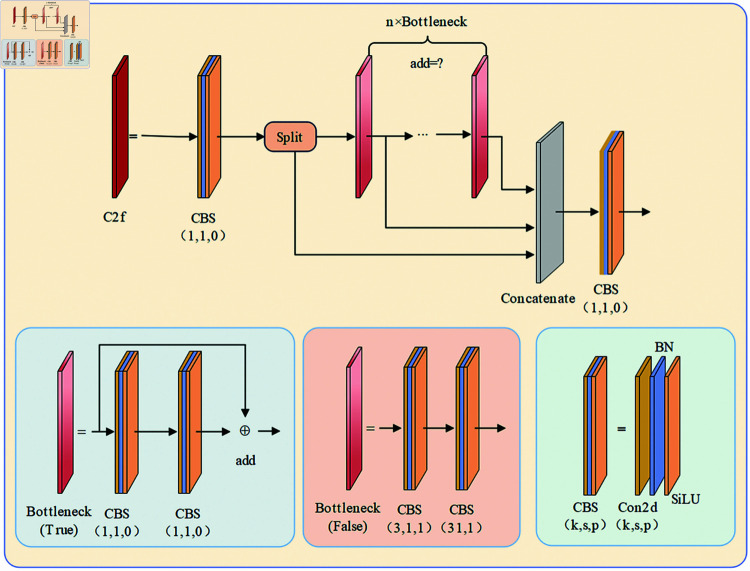
CSPBottleneck with 2 conversions (C2f) module.

The main structure of YOLOv8 can be categorized into Input, Backbone, Neck and Head. Its Backbone follows the CSPDarknet53 structure similar to YOLOv5, and the main structures include CSP Bottleneck with two convolutions (C2F), Spatial Pyramid PoolingFast (SPPF) and Standard convolution with SiLU activation (CBS). In order to obtain higher accuracy and smaller latency, YOLOv8 introduces the C2f module instead of the C3 module in YOLOv5. It combines the advantages of Eﬃcient layer aggregation networks (ELAN) and CSPNet as shown in Fig 2, where ’k’ stands for kernel size, ’s’ for stride, and ’p’ for convolution padding. Inspired by this improved stacking strategy, the C2f module adds several parallel branches, which not only obtains richer information about the gradient flow, but also keeps the model lightweight.

**Fig 3:**
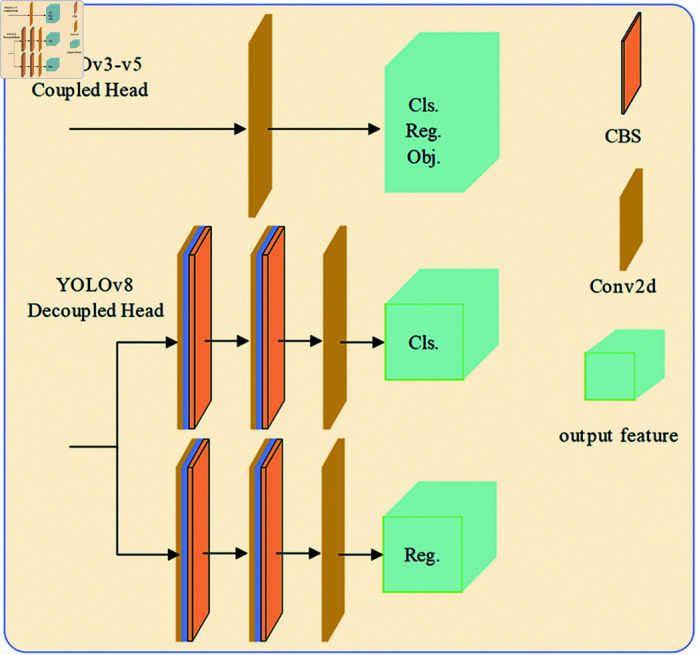
Decoupling head structure.

In the YOLO series of detection algorithms, version YOLOv8 has introduced a decoupled head structure for the first time, as illustrated in Figure 3.This structure decouples the feature mapping, classification prediction, and bounding box regression prediction that were jointly conducted in the head of previous versions into separate prediction tasks. Additionally, this version adopts the Anchor-Free mechanism from YOLOX, predicting the edges of small objects directly and filtering out noise items in the labels. The adjustment of this structure has resulted in a reduction in the number of parameters and the computational complexity, which accelerates the convergence of the model and improves detection accuracy. At the same time, it also enhances the model’s generalization capabilities and robustness.

### Image data acquisition methods

**Fig 4:**
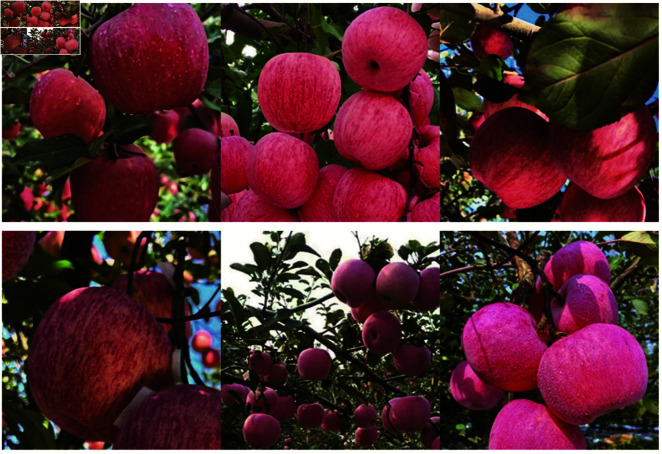
Self-built datasets.

Since there is no unified public dataset related to apple picking, this study uses a self-constructed apple picking dataset. The apple images were mainly from apple orchards in Yantai City, Shandong Province, China, as well as some public datasets on the web. In order to ensure the diversity and authenticity of the dataset, different weather conditions and different orchard environments were taken into account throughout the collection process, and key influencing factors such as shading, illumination, shooting angle, and apple density were fully considered, which improved the reliability and general applicability of the dataset from the source of data collection. Finally, 1586 apple images were collected, and the specific samples are shown in Fig 4.

### Image data processing

**Fig 5:**
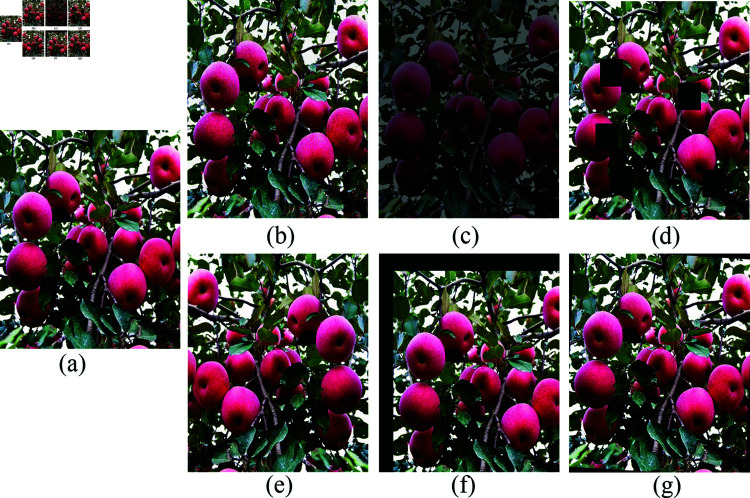
Data enhancement example. (a) original image, (b) add noise, (c) brightness adjustment, (d) add occlusion, (e) mirror, (f) panning, and (g) rotation.

First, to enable the model to learn image features eﬃciently and to expand the dataset, we randomly divide the entire collected dataset into three parts: the training set (1104 images, about 70%), the validation set (316 images, about 20%), and the test set (166 images, about 10%). Second, we performed a series of image preprocessing operations on the original images in the training set to improve the generalisation ability of the training model and reduce the risk of overfitting. The specific processing scheme includes adding noise, adjusting brightness, adding occlusion, mirroring, panning, and rotating, as shown in Fig 5. The final enhanced training set is 9202 images.

**Fig 6:**
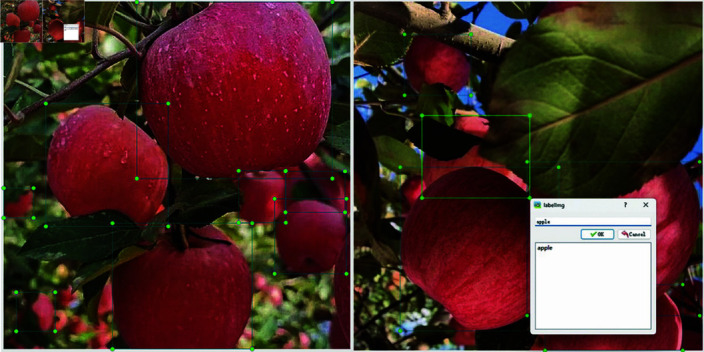
Example of dataset labeling.

In this paper, the open source software LabelImg (version 1.8.6) is used to accurately label the image dataset as shown in Figure 6. During the manual labeling process, the minimum bounding rectangle principle is followed, and a rectangular bounding box is used to label the position of apples in the image. If some parts of the apple are obscured by leaves or other apples, only the visible parts are labeled. This means that the labeling is based on the maximum extent of the visible part of the apple.

## Method

**Fig 7:**
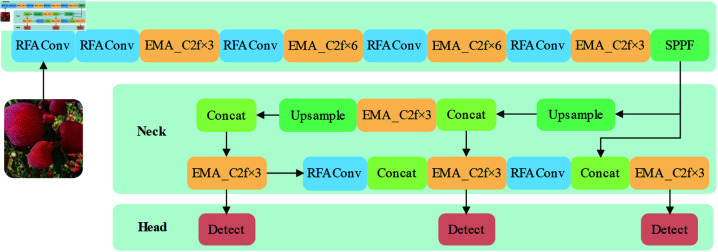
RE-YOLO network structure.

As shown in Fig 7, we propose a RE-YOLO apple picking detection algorithm based on YOLOv8, which is described in detail in this section. Compared to the baseline model, RE-YOLO introduces a Receptive-Field Attention Convolution (RFAConv) that can replace the standard convolution and has higher performance, which can better take into account the differential information of different locations in the image and capture apples of different sizes and locations in the image more effectively [33]. Secondly, we innovatively propose an EMA_C2f module, which is able to detect the subtle differences ignored by traditional models because it refines the image features by emphasizing spatial-channel interactions.The introduction of EMA_C2f enables the model to effectively distinguish the densely distributed apples. Finally, in order to speed up the optimization process and improve the robustness of the model, we propose the use of the Wise Intersection over Union (WIOU) loss function instead of Complete Intersection over Union (CIOU) in the original model.

The blue part of the figure represents the RFAConv module proposed in this paper, which is used to replace the conventional convolutional layers in the backbone and neck parts of the original YOLOv8 architecture.RFAConv improves the accuracy of target detection by making the model more capable of capturing important feature regions through an enhancement strategy that combines the sensory field and the attention mechanism. The orange part represents the EMA_C2f module proposed in this paper, which is used to replace the standard C2f module in the network architecture.EMA_C2f integrates a multi-scale attention mechanism to integrate features at different scales in an eﬃcient manner, and enhances feature transfer and fusion through cross-stage partial convolution to optimise the target detection effect of the model in complex scenes. The detection head remains the same as the original YOLOv8n, but through the synergy between the RFAConv and EMA_C2f modules in the backbone and neck sections, the model significantly improves detection accuracy while maintaining real-time performance. The multi-scale attention mechanism further optimises the model’s adaptability to different sizes, complex backgrounds and occluded targets, achieving higher detection eﬃciency on resource-constrained edge devices. Experiments show that the improved structure outperforms the original YOLOv8n in terms of precision, recall and mAP, especially in densely distributed multi-target scenes.

**Fig 8:**
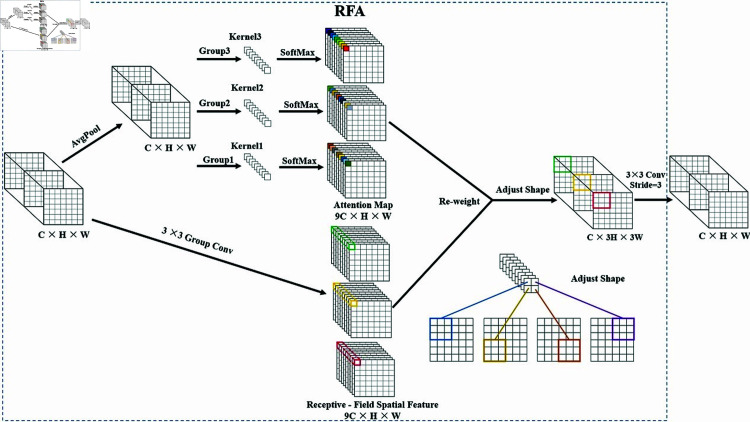
Detailed structure of RFAConv.

### Introduction of receptive-field attention convolution

Currently, spatial attention mechanisms are widely used to improve the performance of convolutional neural networks, such as Convolutional Block Attention Module (CBAM) and Convolutional Block Attention Module (CA). However, the above spatial attention mechanisms only focus on spatial features and do not adequately address the problem of parameter sharing of convolutional kernels. Therefore, this paper introduces a Receptive-Field Attention Convolution that can dynamically adjust the parameters of the convolution kernel. This mechanism not only overcomes the problem that traditional convolution cannot fully consider the differential information of different locations, but also solves the problem of convolutional kernel parameter sharing. It improves the sensitivity and accuracy of the model to detect objects of different sizes, locations and shapes in the image. The method not only emphasizes the importance of different features within the sensory field slider, but also prioritizes the spatial features of the receptive-field.

The RFAConv structure with a 3  ×  3 sized convolutional kernel is shown in Fig 8. Let the input be $X\in R^{C\times H\times W}$. After unfolding, its dimension expands to 9C  ×  H  ×  W, where C, H, and W denote the number of channels, input height, and width, respectively. Moreover, in order to improve the extraction speed of the receptive field space features, RFAConv employs the Group Conv method. That is, in the process of extracting features using a 3  ×  3 convolutional kernel, each 3  ×  3-sized window in the receptive-field space features corresponds to a receptive-field slider.

The core structure of the model consists of a 3  ×  3 Group Convolutional Layer (3  ×  3 Group Conv). This convolutional layer performs an initial convolutional operation on the input features to extract the underlying feature information. These features are then further processed into Receptive-Field Spatial Features, each represented in the form of nine elements (9Cs) and having a spatial dimension of H  ×  W.

This is followed by two key components: the Adjustable Shape and the Attention Map. The Adjust Shape component can be used to adjust the shape or dimension of the features to suit subsequent processing steps. The Attention Map is used to calculate the importance or weight of each feature. Through the attention map, the model can dynamically adjust the contribution of different features to more accurately capture the most important information in the input data.

Finally, the whole structure updates the weights through a reweighting mechanism. In this step, the attention map and the receptive domain features are combined to compute new weights that are used to update the feature map representation. This reweighting mechanism allows the model to dynamically adjust the importance of features according to the characteristics of the input data, thereby improving the model’s expressiveness and adaptability.

In order to minimize the computational cost and the number of parameters, AvgPool is used to aggregate the global information of each receptive-field feature. Then, a 1  ×  1 group convolution is used to interact the information. Finally, SoftMax is used to emphasize the importance of each feature in the receptive-field features. The RFA is calculated as:


$$\begin{array}{ccc} F=\mathrm{Softmax}\left(g^{1\times 1}(\mathrm{AvgPool}(X))\right)\times \mathrm{ReLU}\left(\mathrm{Norm}\left(g^{k\times k}(X)\right)\right)=A_{rf}\times F_{rf}. & & \end{array}$$ (1)

Where $g^{i\times i}$ represents the grouped convolution of size *i* × *i*, *k* denotes the convolution kernel size, *Norm* denotes normalization, *X* denotes the input feature mapping, and *F* is obtained by multiplying the attentional map $A_{rf}$ with the transformed receptive-field spatial feature $F_{rf}$. Compared with CBAM and CA, RFA is able to generate the attention map of a single receptive-field feature.

### Improved EMA_C2f module

**Fig 9:**
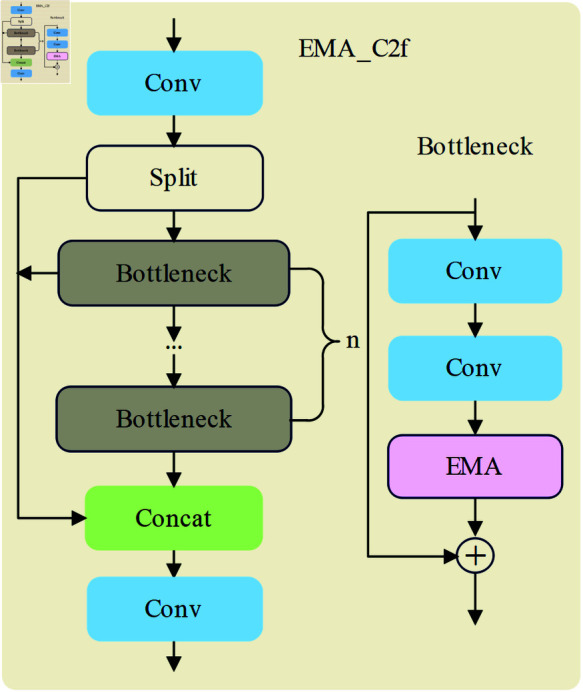
EMA_C2f network structure.

In order to propose complex image features for the apple picking process without reducing the number of parameters of the network structure, we propose an EMA_C2f module as shown in Fig 9. This module combines Eﬃcient Multi-Scale Attention (EMA) with the C2f module to reconstruct some of the channels into batch dimensions and group the channel dimensions into multiple sub-features so that the spatial semantic features are uniformly distributed within each feature group [34].

As can be seen in the figure, EMA_C2f starts with a convolutional layer (Conv), which is responsible for the initial extraction of features from the input data. This convolutional layer is then split by a split layer, i.e. the input data, after passing through the first convolutional layer, is split into two parts, which enter the subsequent processing flow separately. The bottleneck consists of two convolutional layers and an EMA, which are then connected by a concat layer before passing through a convolutional layer for output. The addition of the EMA mechanism allows the network to focus on multiple scales of the input data simultaneously, capturing richer contextual information and feature detail. This mechanism significantly improves the network’s sensitivity and accuracy to the input data.

The EMA module employs an eﬃcient attention mechanism at different scales to selectively integrate and update network parameters. This improvement not only optimizes the eﬃciency of the feature extraction process, but also contributes to the overall improvement of the model performance.

The EMA module employs an eﬃcient attention mechanism at different scales to selectively integrate and update network parameters. This improvement not only optimizes the eﬃciency of the feature extraction process, but also contributes to the overall improvement of the model performance.

**Fig 10:**
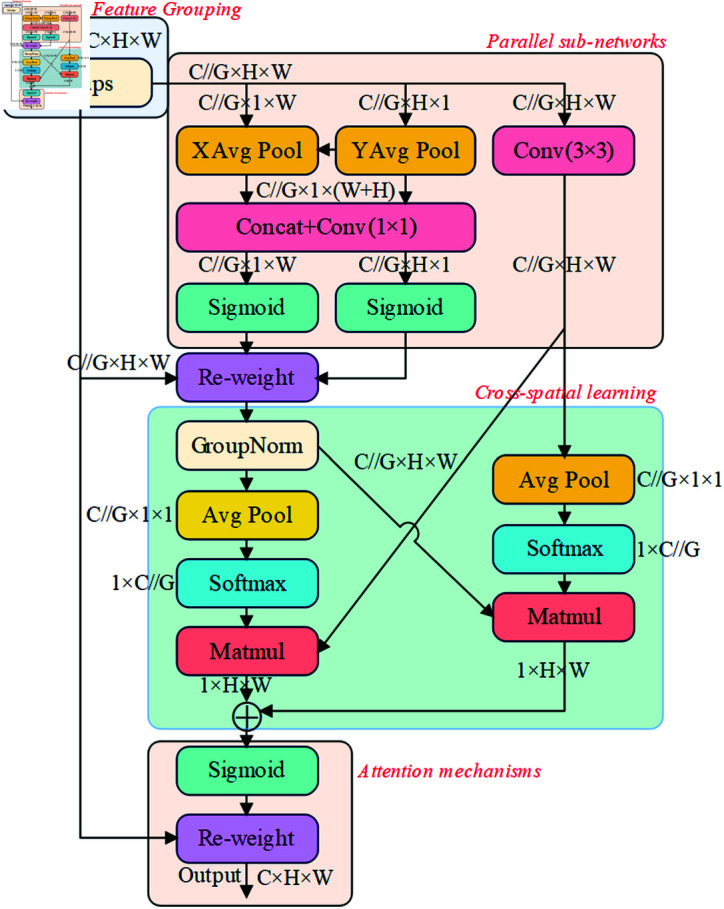
EMA module structure.

Fig 10 depicts the network structure of the EMA module, a novel attentional mechanism based on the traditional coordinated attention (CA) module. It captures the interactions between channels and spatial dimensions to enhance the representation features of an image. The main process includes the following steps:

**Feature grouping:** the EMA module divides the input feature map into multiple sub-features along the channel dimension, which enriches the expressive capability of the model by taking into account the channel correlations and differences to capture different semantic information.

**Parallel sub-network:** EMA module adopts three parallel paths to extract the attention weight descriptors from the grouped feature map. Two of the paths are located in the 1  ×  1 branch, while the third path operates in the 3  ×  3 branch. In the 1  ×  1 branch, the horizontal and vertical channels are encoded using two 1D global averaging pools as shown in Eqs (2) and (3):


$$Z_{C}^{H}(H)=\frac{1}{W}\underset{0\leq i\leq W}{\sum }x_{C}(H,i),$$ (2)

$$Z_{C}^{W}(H)=\frac{1}{H}\underset{0\leq i\leq H}{\sum }x_{C}(j,W).$$ (3)

where *C* denotes the number of input channels, *H* and *W * denote the spatial dimension of the input features, respectively, and $x_{C}$ denotes the input feature of the cth channel.

**Cross-spatial learning:** The EMA module utilizes cross-spatial information aggregation to enhance feature aggregation in different spatial dimensions. For the two spatial attention maps generated by encoding global spatial information in the outputs of the 1  ×  1 and 3  ×  3 branches, the feature maps are first subjected to 2D global adaptive average pooling with reconstruction as shown in Eq (4), and then processed by using the softmax function normalization. Finally, the results of the two branches are multiplied in elemental order to generate the first and second spatial attention maps containing complete spatial location information.


$$\begin{array}{ccc} Z_{C}(H)=\frac{1}{H\times W}\overset{H}{\underset{j}{\sum }}\overset{W}{\underset{i}{\sum }}x_{C}(i,j). & & \end{array}$$ (4)

**Attention mechanism:** After obtaining the spatial attention map from each branch, the EMA module applies a nonlinear Sigmoid function to compute the attention weight values. These values capture the pixel-level pairwise relationships and highlight the pervasive influence of the global context across all pixels. these spatial attention weights are then used to construct the final feature map.

### Improve the loss function

The bounding box regression loss function is a key technique in the field of object detection. This function improves the model’s ability to accurately localize objects by quantifying the deviation between the predicted and actual bounding boxes at different scales. In the case of apple detection, for example, the accuracy and robustness of the detector in dealing with size variations is critical. The sophisticated design of the loss function allows the detection model to self-optimize in recognizing multi-scale objects, adjusting the predicted position with higher accuracy and improving sensitivity to scale differences.

The YOLOv8 model utilizes a CIOU loss function, but it is unable to satisfy both speed and accuracy requirements when evaluating the difference between the predicted and actual bounding boxes [35]. On the contrary, the WIOU loss function optimizes the gradient gain distribution mechanism, which makes the model perform well in capturing targets at different scales and speeds up the model optimization process. Moreover, the WIOU loss function by introducing non-monotonicity, the model treats high and low quality samples equally during training [36]. Therefore, we improve the loss function of RE-YOLO model to WIOU loss function, which significantly improves the accuracy and robustness of the model in multi-scale target detection. The WIOU loss function formula is


$${ℒ}_{\text{WIOU}}=rR_{\text{WIOU}}{ℒ}_{\text{IOU}},\;r=\frac{\beta }{\delta \alpha^{\beta -\delta }},$$ (5)

$$R_{WIOU}=\exp\left(\frac{\left({\left(x-x_{gt}\right)}^{2}+{\left(y-y_{gt}\right)}^{2}\right)}{{\left(W_{g}^{2}+H_{g}^{2}\right)}^{*}}\right)$$ (6)

$${ℒ}_{IOU}=1-IOU.$$ (7)

In the given equations, (*x*,*y*) denotes the center coordinates of the anchor frame; and ($x_{gt}$,$y_{gt}$) denotes the coordinates of the target frame centroid;$W_{g}$ and $H_{g}$ denote the dimensions of the minimum bounding box; and the gradient enhancement factor *r* is dynamically adjusted by the hyperparameters *α*, *δ*, and the nonmonotonic focusing factor *β*. $L_{IOU}$ is the loss function.

where *α* affects the growth rate of the gradient enhancement factor. A larger value of *α* will make the model’s gradient enhancement effect for a particular region more pronounced, improving the model’s sensitivity to the feature region, but too large a value of *α* can lead to over-enhancement of the gradient, affecting the stability of the model. *δ* controls the rate of decay of the gradient enhancement factor. A smaller value of *δ* causes the gradient enhancement factor to decay more slowly during training, which may help the model to better capture long-term dependent features, but may also increase the training time. *β* directly affects the magnitude of the gradient enhancement factor. A larger value of *β* increases the magnitude of the gradient enhancement factor, making the model more effective at gradient enhancement in certain feature regions, thus improving feature extraction.

## Experiment and result analysis

### Evaluation metrics

In order to evaluate the performance of the model objectively, in our experiments, we selected Precision (P), Recall (R) , mean average precision (mAP), Params and frames per second (FPS) metrics to measure the performance of RE-YOLO against other different detection models.

(1) Precision: This metric denotes the proportion of true positive samples within the data classified as positive. The formula for calculation is


$$\begin{array}{ccc} Precision=\frac{TP}{TP+FP}. & & \end{array}$$ (8)

(2) Recall: This metric signifies the proportion of positive samples correctly classified. The formula for calculation is


$$\begin{array}{ccc} Recall=\frac{TP}{TP+FN}. & & \end{array}$$ (9)

(3) F-score represents the harmonic mean of precision and recall, offering a balance between the two. The formula for calculation is


$$\begin{array}{ccc} F-\text{ score }=2\left(\frac{\text{ Recall }\times \text{ Precision }}{\text{ Recall }+\text{ Precision }}\right). & & \end{array}$$ (10)

(4) Mean Average Precision (mAP) is the average precision scores for all classes in object detection tasks. It yields a single figure that characterizes the overall performance of a model across all categories. The formula for calculation is


$$\begin{array}{ccc} mAP=\frac{\overset{N}{\underset{0}{\sum }}Ap_{n}}{N}, & & \end{array}$$ (11)

In this context, *N* represents the total number of categories $AP_{n}$ denotes the average precision for category *n*, corresponding to the area under the Precision-Recall curve. The term mAP@0.5 refers to the mean Average Precision when the IoU threshold is set to 0.5.

(5)Floating point operations per second (FLOPS): Used to evaluate the computational complexity of the model, measured by comparing the number of floating point operations required for the model to perform a single forward propagation, reflecting the computational eﬃciency of the model. The number of parameters is an important criterion for evaluating the simplicity of the model.

(6) Frames per second (FPS): It is an important criterion for evaluating the real-time performance of the model, the higher the FPS, the better the real-time detection performance of the model.

Evaluation indices (5) and (6) are usually used to evaluate the applicability of target recognition models in resource-constrained environments, so this experiment mainly adopts the above evaluation indices to compare and analyse the progress and effect of the RE-YOLO model compared with other models.

### Experimental environment and hyperparameter settings

The experimental setup for this study was configured on a cloud server running Ubuntu 20.04, featuring an Intel(R) Xeon(R) Platinum 8255C CPU, NVIDIA GEFORCE RTX 3080 (10GB) GPU, and 40GB RAM. The training was conducted using PyTorch 2.0, Python 3.8, and CUDA 11.8, with carefully selected hyperparameters to optimize the model’s performance. The number of training epochs was set to 200, during which Mosaic data augmentation was disabled in the last 10 epochs to prevent overfitting and stabilize the model for better generalization. The batch size was set to 15, balancing memory usage and convergence stability. An initial learning rate of 0.0001 was chosen to ensure gradual and stable optimization, dynamically adjusted using a cosine annealing scheduler to enhance convergence. A momentum value of 0.937 was used to smooth gradient updates and accelerate convergence, while weight decay was set to 0.0005 to regularize the model and reduce overfitting risks. The optimizer was configured to adapt dynamically based on training dynamics, improving the optimization process. These hyperparameters were tested and refined to achieve a balance between stability, convergence speed, and model generalization, contributing significantly to the high precision, recall, and mAP achieved in our experiments. These settings were consistently applied to all comparative models, ensuring a fair performance evaluation. By systematically fine-tuning the training parameters, the proposed RE-YOLO algorithm demonstrated robust and eﬃcient performance, suitable for real-world apple-picking applications.

### Ablation experiments

In order to evaluate the impact of the three improvement schemes on the model performance, we conducted ablation experiments on a self-constructed dataset, while keeping the training parameters the same. In this experiment, we focus specifically on the performance of the RE-YOLO model in terms of recall and precision. Recall measures the number of targets correctly identified by the model, while precision indicates how many of the targets identified by the model are correct. Maintaining a balance between the two is critical in target detection tasks, especially in dense fruit occlusion and complex lighting environments. The experimental results are shown in Table 1.

**Table 1:** Results of ablation experiment.

YOLOv8n	RFAConv	EMA_C2f	WIOU	P/%	R/%	mAP@0.5/%	mAP@0.5-0.95/%
*✓*				90.8	80.5	86.9	58.9
*✓*	*✓*			89.6	82.4	88.4	59.9
*✓*		*✓*		85.8	80.9	87.5	59.4
*✓*			*✓*	86.7	83.4	88.7	59.6
*✓*	*✓*	*✓*		91.2	82.6	89.6	60.6
*✓*	*✓*		*✓*	89.3	81.9	88	58.8
*✓*	*✓*	*✓*	*✓*	92.8	82.6	89.6	62.8

First, we fused each of the above three modification schemes into the baseline model YOLOv8n for experiments. Although the precision is reduced, the recall is improved by 1.9%, 0.4%, and 2.9%, respectively; mAP@0.5 is improved by 1.5%, 0.6%, and 1.8%, respectively; and mAP@0.5-0.95 is improved by 1%, 0.5%, and 0.7%, respectively. After adding the WIOU improvement scheme alone, the precision and recall of the model show a more substantial improvement, which demonstrates the effectiveness of the WIOU loss function improvement scheme. The above experimental results fully demonstrate that all three improvement schemes can significantly improve the model performance.

Second, two of the improvement schemes are combined and fused into the baseline model for experimentation. When the RFAConv and EMA_C2f modules are fused, the precision, recall, mAP@0.5, and mAP@0.5-0.95 metrics improve by 0.4%, 2.1%, 2.7%, and 1.7%, respectively. After incorporating the EMA_C2f module and the WIOU loss function, the above metrics improve by 0.3%, 1.4%, 1.1%, and 0.4%, respectively. The results indicate that the combination of the two improvement schemes still significantly contributes to the model’s performance.

After combining the above three strategies, our proposed RE-YOLO model improves 2%, 2.1%, 2.7% and 3.9% in terms of precision, recall, mAP@0.5, and mAP@0.5-0.95, respectively. These results confirm the substantial improvement in RE-YOLO assay performance. Moreover, the number of parameters and computational complexity of RE-YOLO (GFLOPs  =  8.7) increased only slightly compared to the baseline model (GFLOPs  =  8.1). The slight increase in complexity is justified by the significant improvement in recall and mAP, demonstrating the model’s excellent ability to detect targets more accurately and consistently under a variety of conditions. The final experimental results show that our improved scheme is effective and significantly improves the performance of the model for the task of target recognition in apple picking.

**Fig 11:**
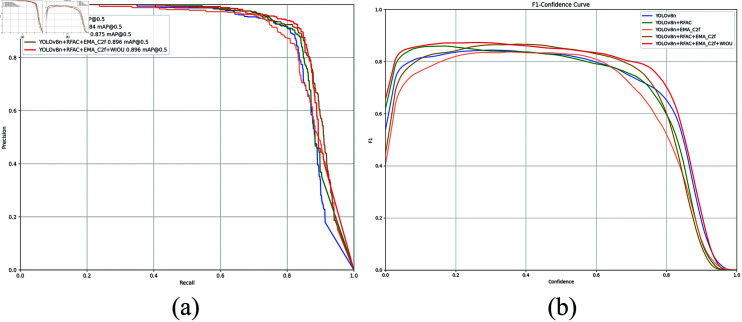
F1-score and precision recall (PR) curves during the ablation experiment training process.

Fig 11 illustrates the F1-score curve and PR curve from the ablation experiment. As evident from the figure, the PR curve and F1-score curve of RE-YOLO surpass the others significantly, indicating that our proposed model achieves higher precision with the same recall and maintains a balance between precision and recall under various thresholds. This further confirms the effectiveness of the RE-YOLO model in eﬃciently recognizing targets and reducing false alarms in target detection tasks.

**Fig 12:**
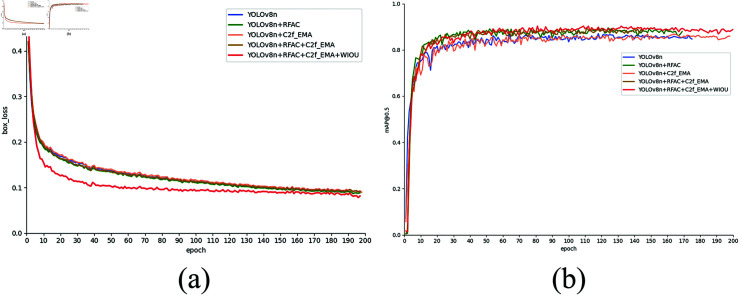
Box_loss curves and mAP0.5 curves during training.

Fig 12 displays the box_loss curve and mAP@0.5 curve throughout the training process. It’s evident from the graph that the box_loss curve decreases significantly faster compared to other models, indicating the stronger optimization capability of our proposed model concerning target box loss, enabling it to reach the target state more rapidly. Furthermore, the mAP@0.5 curve also surpasses that of other models, suggesting that our model exhibits higher accuracy in target detection, particularly at lower confidence thresholds. These findings further underscore the superior performance and reliability of our proposed model in target detection tasks.

### Comparison experiments with the baseline model

**Fig 13:**
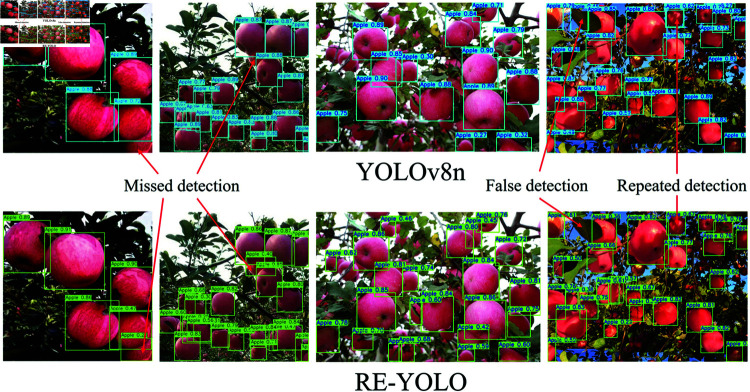
RE-YOLO vs. baseline model for comparison of test results.

In order to verify the performance difference between the proposed RE-YOLO model and the baseline model, we utilize the trained models from the ablation experiments for the target detection experiments, and the specific results are shown in Fig 13.

The test images shown in the figure are images that we have retaken. In the figure, the first row displays the detection results of the baseline model, while the second row presents the detection results of the RE-YOLO model. It is evident from the figure that our proposed model exhibits higher accuracy and robustness in the apple detection task compared to the baseline model. The baseline model suffers from missed detections, false detections, and repeated detections, whereas our model excels in recognizing the targets in the image and significantly reduces false positives and repeated detections during the detection process. These experimental results further validate the superior performance of our proposed model in the target detection task and underscore its potential and reliability in practical applications.

### Comparative experiments with other models

To further validate the superiority of the RE-YOLO model, we conducted a comparative test of several commonly used target detection models under the same experimental conditions. The performance evaluation metrics are mainly based on mAP@0.5, floating point operations per second (FLOPS), number of parameters (Params), model size and frames per second (FPS). The experimental results are presented in Table 2.

Firstly, the YOLO series algorithms outperform the other three algorithms significantly in terms of image processing speed and number of parameters, making them more suitable for deployment on high-speed running robots. Additionally, our proposed RE-YOLO algorithm also demonstrates obvious advantages over YOLOv5n and YOLOv6n.Secondly, in terms of the precision of the algorithm, the RE-YOLO algorithm achieves the highest performance compared to other algorithms. It shows a 1.93% improvement in mAP@0.5 compared to the YOLOv5n algorithm, 5.7% compared to YOLOv6n, and 3.2% compared to YOLOv7-Tiny.

These comparisons further aﬃrm the effectiveness of RE-YOLO in the target detection task of apple picking robots, and also demonstrate the effectiveness of our improved scheme.

## Conclusions

In this paper, we propose an apple-picking recognition algorithm called RE-YOLO to address the target recognition challenges faced by today’s apple-picking robots, including dense distribution, occlusion, and degradation of recognition accuracy due to illumination variations.

The algorithm is optimised in several ways. First, we innovatively introduce the RFAConv module to replace the standard convolutional module in the YOLOv8n network architecture. This solves the problem of sharing the parameters of the convolutional kernel and significantly improves the model’s ability to capture information about the differences between different locations in the image, ensuring rich and accurate feature extraction from the image. Second, we propose an EMA_C2f module. By introducing the EMA attention mechanism, the spatial semantic features of an image are uniformly distributed within different feature groups, enabling the model to capture both short-range and long-range image features, and significantly improving the model’s ability to detect densely distributed apples. Finally, the WIOU loss function is introduced during training, which not only speeds up the overall optimisation process of the model, but also effectively captures targets of different sizes.

**Table 2:** Results of comparative experiments with different models.

Model	mAP@0.5(%)	FLOPS(G)	Params(M)	Model size(MB)	FPS
Fast R-CNN	58.3	78.12	41.1	113.6	4.3
SSD	82.8	137.09	23.8	96.7	9.8
RetinaNet	76.7	81.69	36.1	146	4.6
YOLOv5n	87.9	4.2	1.76	3.9	65.4
YOLOv6n	84.8	11.4	4.7	10	87.1
YOLOv7-Tiny	86.8	13	6.01	12.3	153.1
YOLOv8n	86.9	8.1	3.01	5.96	133.3
RE-YOLO	89.6	8.7	3.1	6.4	123.5

The experimental results confirm the effectiveness of these optimisations. The introduction of RFAConv improves the model’s ability to discriminate features in regions of high object density, addressing the occlusion problem. Similarly, the EMA_C2f module balances feature extraction across different image scales, which is critical for detecting densely clustered targets. The WIOU loss function contributes to better localisation accuracy for multi-scale targets, improving both precision and recall. Together, these components ensure that the RE-YOLO model performs robustly in complex natural environments, including varying lighting conditions and dense foliage.

In our next work, we will deploy the model in a robotic system for testing and improvement, to provide a high performance detection model for automated fruit picking. By conducting field experiments and integrating the detection algorithm with robotic systems, we aim to refine the real-time performance and further improve the robustness and applicability of the system in practical scenarios.
